# Modulation of Sex Pheromone Discrimination by a UDP-Glycosyltransferase in *Drosophila melanogaster*

**DOI:** 10.3390/genes11030237

**Published:** 2020-02-25

**Authors:** Stéphane Fraichard, Arièle Legendre, Philippe Lucas, Isabelle Chauvel, Philippe Faure, Fabrice Neiers, Yves Artur, Loïc Briand, Jean-François Ferveur, Jean-Marie Heydel

**Affiliations:** 1 Centre des Sciences du Goût et de l’Alimentation, AgroSup Dijon, CNRS, INRAE, Université Bourgogne Franche -Comté, F-21000 Dijon, France; stephane.fraichard@u-bourgogne.fr (S.F.); ariele_l@hotmail.com (A.L.); isabelle.chauvel@u-bourgogne.fr (I.C.); philippe.faure@u-bourgogne.fr (P.F.); fabrice.neiers@u-bourgogne.fr (F.N.); yves.artur@u-bourgogne.fr (Y.A.); loic.briand@inra.fr (L.B.); jean-francois.ferveur@u-bourgogne.fr (J.-F.F.); 2 Institut d’Ecologie et des Sciences de l’Environnement de Paris, F-78000 Versailles, France; philippe.lucas@inra.fr

**Keywords:** olfaction, chemosensory, odorant-degrading enzymes, perireceptor, drosophila

## Abstract

The detection and processing of chemical stimuli involve coordinated neuronal networks that process sensory information. This allows animals, such as the model species *Drosophila melanogaster*, to detect food sources and to choose a potential mate. In peripheral olfactory tissues, several classes of proteins are acting to modulate the detection of chemosensory signals. This includes odorant-binding proteins together with odorant-degrading enzymes (ODEs). These enzymes, which primarily act to eliminate toxic compounds from the whole organism also modulate chemodetection. ODEs are thought to neutralize the stimulus molecule concurrently to its detection, avoiding receptor saturation thus allowing chemosensory neurons to respond to the next stimulus. Here, we show that one UDP-glycosyltransferase (UGT36E1) expressed in *D. melanogaster* antennal olfactory sensory neurons (OSNs) is involved in sex pheromone discrimination. UGT36E1 overexpression caused by an insertion mutation affected male behavioral ability to discriminate sex pheromones while it increased OSN electrophysiological activity to male pheromones. Reciprocally, the decreased expression of UGT36E1, controlled by an RNAi transgene, improved male ability to discriminate sex pheromones whereas it decreased electrophysiological activity in the relevant OSNs. When we combined the two genotypes (mutation and RNAi), we restored wild-type-like levels both for the behavioral discrimination and UGT36E1 expression. Taken together, our results strongly suggest that this UGT plays a pivotal role in Drosophila pheromonal detection.

## 1. Introduction

The initiation of olfaction is mediated by olfactory receptors which interact with a variety of odorant molecules allowing their fine detection and discrimination. Receptor activation triggers the olfactory signal transmitted as a depolarization train of spikes to the central nervous system, this resulting in its integrated perception and an adapted behavioral response. The detection of odorants is modulated by perireceptor events through which a complex series of biochemical processes are carried out to influence the entry, exit and/or residence time of odorant molecules in the receptor environment [1,2]. Perireceptor mechanisms play a significant role in the modulation of the stimulus availability for receptors, therefore impacting its reception and subsequent perception. They include, first, the binding and transport of hydrophobic odorant molecules by odorant-binding proteins (OBPs) [3,4,5] and then their inactivation and elimination by detoxification enzymes called odorant- metabolizing enzymes in vertebrate (OMEs) [6] or odorant-degrading enzymes (ODEs) in insect [2,7]. Indeed, some metabolizing enzymes can be expressed, either specifically, and/or at a high concentration, in the olfactory tissues or organs [6,8,9,10,11]. These enzymes are primarily involved in detoxification processes by catalyzing the biotransformation of hydrophobic xenobiotic molecules through two phases often but not necessarily successive. During phase I, functionalization enzymes introduce, or unmask, functional groups into xenobiotics through oxidation, reduction or hydrolysis reaction (e.g., cytochrome P450, CYP; aldehyde dehydrogenase; carboxylesterase, CES). During phase II, functionalized metabolites can be conjugated to hydrophilic products by transferases enzymes such as UDP-glycosyltransferases (UGT) or glutathione transferases (GST). Thus, the resulting inactive hydrophilic metabolites can be easily eliminated. These enzymes are organized in networks allowing to metabolize a broad range of substrates.

In insects, the characterization of metabolizing enzymes received an increasing interest with regard to their role in insecticide resistance, adaptation to host plant volatile and their function; as ODEs, in the termination of the olfactory signal to maintain a relatively high olfactory sensitivity toward new stimuli. In particular, recent studies investigating the antennal transcriptome in different species identified varied ODEs including CYP, CES, GST and UGT [11,12,13,14,15,16,17,18,19,20,21,22,23,24]. These reports have completed and confirmed the case-by-case identification of previously characterized ODEs [25,26,27,28,29,30,31]. Altogether, a high number of diverse antennal ODEs have been identified including among others, 30 CES and 84 CYP in *Spodoptera littoralis* [14,26,27,32,33], and 31 GST and 57 CYP in *Drosophila melanogaster* antennae [11]. 

However, data about ODE function in chemosensory process are still limited, likely because of (i) the current focus on odorant/receptor interaction and (ii) the complexity due to the high diversity of enzymes and odorant substrates in different species and strains. Phase I ODE’s function was initially investigated with regard to the perception of pheromones [2,34,35] because of their critical role and their ability to trigger specific and measurable sexual behavior [7,36]. Although phase II enzymes are expected to play a similar role as phase I enzymes in the metabolism of plant volatiles, pheromones or diverse odorants [9,16,20,22], no olfactory function was revealed so far in insects. Among phase II ODEs, the study of UGTs received an increasing interest during the last decade, especially with regard to insecticide resistance and plant defense mechanisms [37,38,39,40,41,42,43,44]. This major class of enzymes in the animal kingdom [45,46] catalyzes in insects the conjugation of a glycosyl group brought by a UDP-glycoside to hydrophobic substrates [47,48]. The fact that UGT expression is enriched in antennae supports its potential role in olfaction [11,47,49,50,51], in relation with its high number of isoforms: 20 in *Holotrichia parallela Motschulsk*y and 11 in *S. littoralis* and 19 in *D. melanogaster* [11].

In the present study, we combined molecular, genetic, behavioral and electrophysiological approaches to investigate the influence of a phase II ODE, a UGT (UGT36E1), on the ability of *D. melanogaster* males to discriminate sex pheromones. UGT36E1 (CG17322) is one of the most expressed UGT in *D. melanogaster* [47]. We used both mutational and interferential RNA approaches to target UGT36E1 expression in the fly olfactory tissues. We found that both the decreased and increased UGT expression in the peripheral olfactory system affected sex pheromone discrimination in a reciprocal manner.

## 2. Materials and Methods

### 2.1. Stocks and Flies

All *D. melanogaster* strains were raised on yeast/cornmeal/agar medium and kept at 24 ± 0.5 °C with 65 ± 5% humidity on a 12L:12D cycle. The wild-type Dijon2000 strain (Di2), used as a control, has been maintained in our lab for more than a decade. The P-UGT36E1 mutant strain (y1 w67c23); P{SUPor-P}CG17322KG04070 (#13518) and the transgenic strains *neur*-GAL4 (#6393); *Orco*-GAL4 (#23292); UAS-CD8::GFP (#5130) were obtained from the Bloomington Drosophila Stock Center (Indiana University). The *Gr66a*-GAL4 strain was kindly provided by Dr. Hubert Amrein. RNAi transgenics against UGT36E1 were purchased from the VDRC [52]. To isogenize its genetic background, the P-UGT36E1 mutation was outcrossed to the Di2 wild-type genetic background of the w^1118^ strain by five successive backcrosses [53]. Crosses were performed using standard techniques and genetic methods [54].

### 2.2. Behavior

The behavior of single tester males towards two Di2 headless target flies (a male and a female) was measured over a 5 min period. All flies were isolated 0–4 h after eclosion under CO_2_ anaesthesia. A few minutes prior to the test, anaesthetized target flies were decapitated with a razor blade (cleaned with ethanol between each sex). In all courtship tests, we used headless flies which do not copulate: therefore, decapitation allows courtship duration to be standardized. Moreover, some tests were performed under red light (25W with a Kodak safe-light filter no. 1) under which flies are virtually blind. In these conditions, most behavioral, visual and acoustic variables associated with the object fly are removed, this enhancing the behavioral effect of pheromones [55,56]. Only upright headless flies were used for the test. Tester males were individually aspirated (without anaesthesia) under a watch glass used as an observation chamber (1.6 cm^3^). After 5 min, the two headless target flies were simultaneously introduced and the courtship index (CI) toward each target was measured (CIf = courtship towards female flies; CIm = courtship towards male flies). CI corresponds to the fraction of the time spent courting relative to the total amount of time multiplied by 100. We only took in account male active courtship (wing vibration, licking and attempting copulation; the very brief episodes of tapping behavior were rarely seen under red light and thus was not included in the CI calculation). No qualitative difference was noted between the different courtship sequences shown by the different tester male genotypes. The discrimination measure was based on the comparison between CIf and CIm for each genotype and condition used. *n* > 30. Locomotor activity, resulting of the cumulation of the activity of single flies, was measured during five periods of 10 s (total of 50 s), over five minutes (*n* > 24). All tests took place 1–5 h after lights on, in a room at 24 ± 0.5 °C with 65 ± 5% humidity. Tests were performed over several days to randomize the experimental variation of uncontrolled environmental parameters.

### 2.3. q-PCR

To process fly tissues, whole frozen flies were vortexed over two superposed meshes of different calibers. From bottom to top, tissue fractions successively consisted of the appendages (maxillary palps, antennal segments), heads (without appendages), and bodies (without heads or appendages). RNAs were extracted from *Drosophila*-fractionated tissues using RNAzol reagent (Euromedex, Souffelweyersheim, France) and treated with RNase-free DNase to avoid contamination by genomic DNA. Total RNA (1 µg) was reverse-transcribed using the iScript cDNA Synthesis Kit (BioRad, Hercules, USA). q-PCR reactions were carried out on a MyIQ (BioRad, Hercules, USA) using the IQ SYBR Green Supermix (BioRad, Hercules, USA). The q-PCR conditions were as follows: 98 °C for 5 min to activate the hot-start DNA polymerase, followed by 40 cycles of 95 °C for 30 s, 65 °C for 30 s and 72 °C for 30 s. Each reaction was performed in triplicate and the mean of the three independent biological replicates (corresponding to three extractions) was calculated. All results were normalized to the RP49 and Actine5C mRNA level and calculated using the ΔΔCt method [57].

### 2.4. Expression Pattern of GAL4 Strains

The expression pattern of all GAL4 strains was always observed in F1 males resulting of the cross between UAS-GFP females and GAL4 males. F1 males were dissected and analyzed using fluorescent microscopy (Leica DM5000B, Leica Microsystems, Wetzlar, Germany). Frozen sections of antennae were collected and fixed in 4% formaldehyde in PBS for 30 min and washed with PBS. Antennal sections were then stained with goat anti-GFP (1:500; Rockland, Limerick, Ireland) and mouse anti-Elav (1:1000; Developmental Studies Hybridoma Bank). Detection of the different primary antibodies was carried out using AlexaFluor488 anti-goat (1:800, Molecular Probes, Eugene, USA) and AlexaFluor594 anti-mouse (1:800, Molecular Probes, Eugene, USA). After mounting in Vectashield (Vector Labs, Burlingame, CA, United States) images were made with a Leica TCS-SP2 confocal microscope. 

### 2.5. Electrophysiology

Electroantennograms (EAGs) were recorded with Ringer-filled capillary glass electrodes (NaCl 120 mM; KCl 5 mM; CaCl_2_ 1 mM; MgCl_2_ 4 mM; HEPES 10 mM; pH 7.2) from 4- to 6-day-old adult male flies [58]. Immobilized flies were placed in a 1000 µL micropipet-tip, the extremity of the tip was filled with cotton and flies were blocked by dental wax. The ground electrode (tip diameter = 2–3 µm) was introduced into the right eye and the recording electrode (tip diameter = 10 µm) was placed on the distolateral surface of the third antennal segment. Recorded signals were amplified (×100) and low-pass filtered at 1 kHz using an Axopatch 200B amplifier (Molecular Devices, San José, USA) and monitored on a computer using a Digidata 1440A acquisition board (Molecular Devices, San José, USA) driven by Clampex 10 software (Molecular Devices, San José, USA). 

Odorants were diluted 1/1000 (v/v) in paraffin oil and delivered from Pasteur pipettes containing 10 µL of diluted odorant deposited on a strip of filter paper. Stimulation with sex-specific odors was obtained by blowing a flow of humidified air (37.5 mL/s) for 1 s through a Pasteur pipette (inner diameter 5 mm, length 250 mm) containing 25 same-sex flies (4- to 6-day-old) [59]. Flies were placed in the tube one hour prior to experiments. 

Flies were first stimulated with 2-Heptanone (2-H), then with odors of living males and finally with odors of living females. Flies were allowed to recover at least 2 min between each stimulation. The odorant 2-H molecule, which elicits a robust electrophysiological response, was chosen to normalize the EAG responses to male and female odors.

### 2.6. Statistical Analysis

For q-PCR, transcript level ratios were compared between strains (or body fractions) using Relative Expression Software Tool [57] (REST, REST-MCS beta software version 2) with 2000 iterations. This is based on the probability of an effect as large as that occurring under the null hypothesis (no effect of the treatment), using a randomization test (Pair-Wise Fixed Reallocation Randomization Test) [60]. For behavior and EAG, statistical analyses were performed using XLSTAT software. Within each genotype, the CI directed towards each sex target was compared with a Student’s *t*-test. Since CIf and CIm are linked, they were compared between strains using ANOVA and LSD-Fischer post-hoc tests (sex target/strain genotype). Locomotor activity values were normally distributed and compared with a Mann-Whitney test (*p* < 0.05). The difference of EAG amplitude between stimulation was compared using a Wilcoxon bilateral test. EAG comparison between strains was performed with a Kruskal-Wallis test (*p* < 0.05).

## 3. Results/Discussion

**Figure 1:**
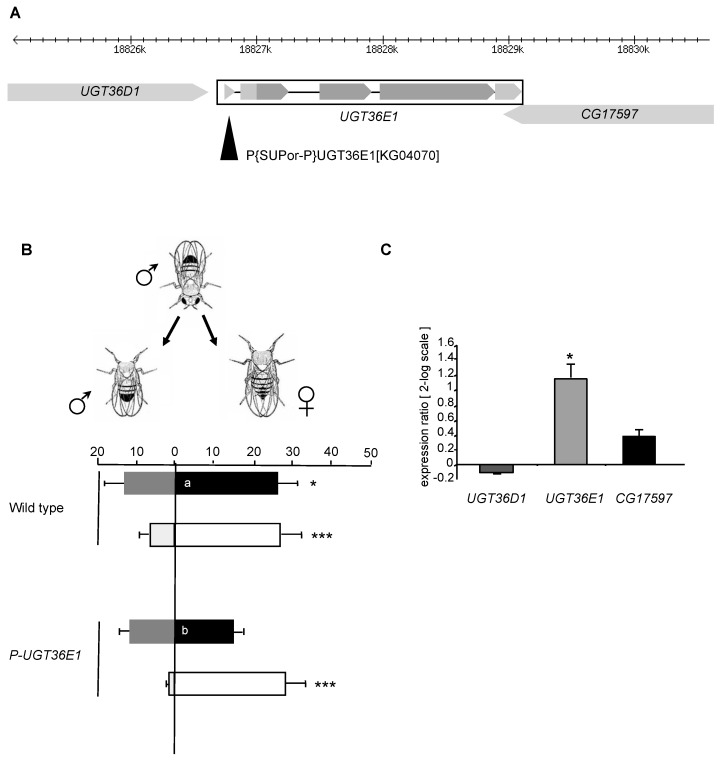
Effects of the P-element insertion in UGT36E1. (**A**) Schematic organization of the 37B1 chromosomal region. Arrowhead indicates the position of the P-element inserted in the 5’UTR region of UGT36E1. (**B**) The P-element inserted in UGT36E1 (P-UGT36E1) affects sex pheromone discrimination in male flies. Tests were carried out either under red light (filled bars) or white light (empty bars). Each mirrored bar represents the mean (± s.e.m.) courtship index towards female (CIf) and male (CIm). Individual 4-day-old tester males directed towards female (right) and male (left) headless targets, simultaneously presented during a 5 min observation period. Tester males were homozygous (P-UGT36E1) for the P-element mutation, or wild-type (Dijon strain); target flies always belonged to this wild-type strain. Significant differences for male ability to discriminate between the two sexes are shown next to each mirrored bar as ***: *p* < 0.001; **: *p* < 0.01; *: *p* < 0.05 (Student’s *t*-test). Courtship data towards each sex were tested using ANOVA and LSD Fisher tests (letters within bars indicates significant differences towards each sex). For each test, the number (n) was *n* > 40 (under red light) and *n* > 30 (under white light). (**C**) The P-element insertion affects RNA expression levels of UGT36E1. The expression levels of UGT36E1, UGT36D1 and CG17597 were analyzed by real-time PCR. The significance of differences in the ratio of transcript levels was based on a comparison between wild-type and P-UGT36E1 homozygous mutant flies (in log_2_ scale control = 0). Data represent the mean (± s.e.m.) of the expression ratio (mutant: wild-type) carried out with three independent extractions.

We investigated in vivo the involvement of ODEs in the discrimination of sex pheromones in *D. melanogaster*. In this species, female and male pheromones diverge and induce reciprocal effects: they tend to stimulate or to inhibit male courtship behavior, respectively [61]. We discovered that a transposable P-element (P-UGT36E1) inserted into the UGT36E1 gene affected male discrimination of sex pheromones (Figure 1A). To determine the ability of single tester males to discriminate sex partners, we measured their courtship intensity (or courtship index = CI) toward both female and male target flies simultaneously presented [55]. This paradigm allowed us to measure their CI towards a female target fly (CIf) and towards a male (CIm). The CIf/CIm comparison allowed us to determine the ability of tester males to discriminate the two sex targets, for each genotype and experimental condition. We compared male discrimination ability under red light (Figure 1B; filled bars), in which visual stimuli provided by both target flies are ineffective and under white light (empty bars) allowing tester males to discriminate sex target based on their different morphology. Under red light, P-UGT36E1 homozygous mutant males showed similar CIm and CIf. In particular, mutant males showed a decreased CIf compared to wild-type tester males (*p* = 0.0045), while their CIm was not affected (*p* = ns). The loss of sex discrimination was likely due to a chemosensory defect given that mutant males tested under white light showed a strong preference to the female target, similarly to wild-type males (Figure 1B). This indicates that the mutation did not affect its overall sexual activity; moreover, the male ability to use visual cues to discriminate sexual partners is functional. Therefore, these results suggest that the P-UGT36E1 mutation affects the ability of male flies to discriminate sex pheromones.

Given that the P-element inserted upstream of the UGT36E1 gene is also in the vicinity of two other genes (UGT36D1, CG17597; Figure 1A), we measured mRNA expression of the three genes in P-UGT36E1 mutant males. Quantitative RT-PCR (q-PCR) revealed that only UGT36E1 significantly changed its mRNA expression (>two-fold increase in P-UGT36E1 flies as compared to controls; Figure 1C). The absence of any significant variation in expression between the head, thorax, abdomen, and appendages of either wild-type flies or mutant flies suggests that this UGT has not a tissue-specific expression (Figure S1). Moreover, this ubiquitous mutation-induced increase of UGT expression in P-UGT36E1 flies had no general behavioral effect given that both locomotor activity and global courtship index (CIf + CIm) were similar to those of wild-type flies under white light (Figure S2; Figure 1B). 

**Figure 2:**
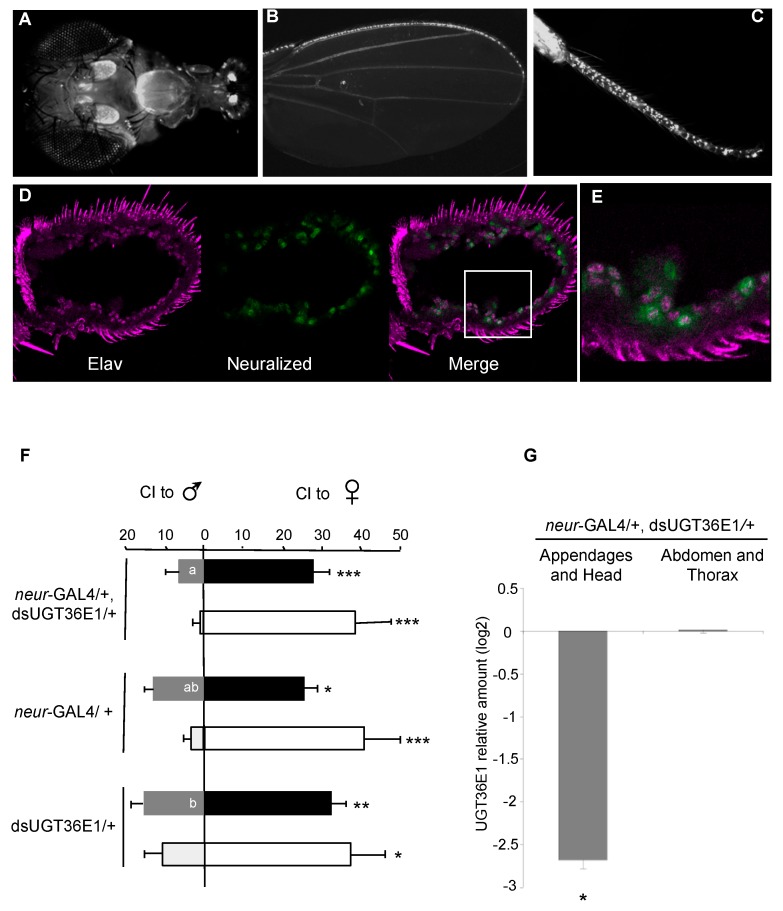
Effect of dsUGT36E1 in the peripheral chemosensory system. A strong expression of *neuralized*-GAL4 (*neur-*GAL4) was detected in (**A**) the antennae and the proboscis, (**B**) wing margins, (**C**) legs of adult flies. (**D**) Antenna stained for Elav protein (red) and anti-GFP (*neur*-GAL4). (**E**) Magnified view of antennal neurons expressing Elav and *neur*-GAL4. (**F**) Male ability to discriminate sex partners in “*neur-*GAL4/+, dsUGT36E1/+” testers and in both transgenic controls (*neur-*GAL4/+ and dsUGT36E1/+) under red light (filled bars) and white light (empty bars). For each test, *n* > 35. (**G**) Real-time PCR analysis showing UGT36E1 mRNA level in different tissues of “*neur-*GAL4/+, dsUGT36E1/+” flies. The significant difference in transcript level ratio is based on a comparison between control (dsUGT36E1/+) and “*neur-*GAL4/+, dsUGT36E1/+” genotypes (pair-wise fixed reallocation randomization test). For statistics and conditions, see Figure 1. *** *p* < 0.001; ** *p* < 0.01; * *p* < 0.05.

To expand our investigation on the behavioral effect of UGT36E1, we used RNAi targeted against UGT36E1 (UAS-dsUGT36E1; hereafter dsUGT36E1) to knock down UGT36E1 RNA expression. We targeted dsUGT36E1 distinct subsets of tissues using several GAL4 drivers. We first used the *neuralized*-GAL4 (*neur-*GAL4) driver to target adult chemosensory organs involved in the perception and processing of pheromones (antenna, proboscis, wing margin and tarsa; Figure 2A–C). We found that *neur*-Gal4 is expressed in antennal neuronal cells (Figure 2D,E). Under red light, experimental males (“*neur-*GAL4/+, dsUGT36E1/+”) showed increased sex discrimination compared to both control parental transgenic genotypes (*neur-*GAL4/+ and dsUGT36E1/+; Figure 2F). This effect was mostly due to the lower CIm shown by knockdown flies (Cim = 6.5 vs. 16.5 for dsUGT36E1/+ control males; *p* = 0.022). However, CIf were similar in mutant and control tester males (CIf = 28 and 32, respectively). Moreover, *neur-*GAL4/+, dsUGT36E1/+ males showed a 6-fold decrease for mRNA expression level in sensory appendages and heads compared to dsUGT36E1/+ controls (*p* = 0.031; Figure 2G). Differently, no expression difference was detected between the abdomen and thorax of the two male genotypes. 

**Figure 3:**
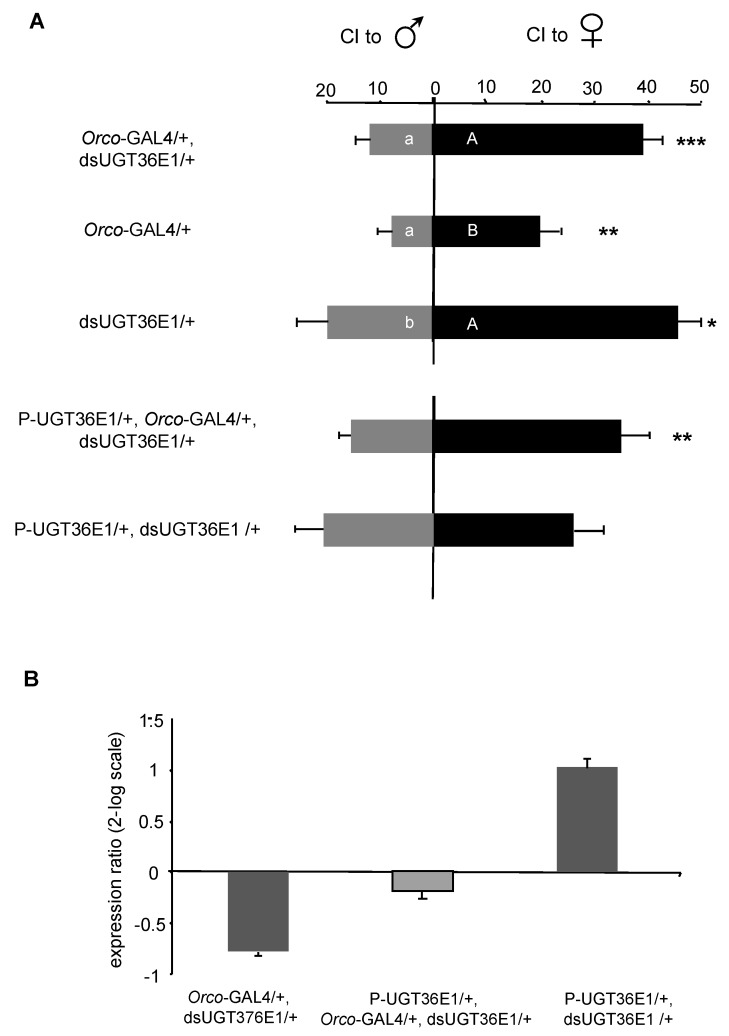
Expression and effect of dsUGT36E1 targeted in various sensory neurons subsets. (**A**) Targeting the dsUGT36E1 transgene in most olfactory sensory neurons with the *Orco*-GAL4 transgene (“*Orco*-GAL4/+, dsUGT36E1/+”) improved mate choice performance compared to both transgenic controls. A similar targeting of this transgene in the P-UGT36E1 mutant background (in “*Orco*-GAL4/+, dsUGT36E1/+”, but not “P- UGT36E1/+, dsUGT36E1/+”) rescued male performance. For each behavioral test, *n* > 35. All courtship tests were carried out under red light. (**B**) Quantitative analysis of UGT36E1 expression in the sensory appendages of “P-UGT36E1/+, *Orco*-GAL4/+, dsUGT36E1/+” flies compared to “*Orco*-GAL4/+, dsUGT36E1/+” and “P-UGT36E1/+, dsUGT36E1/+” control flies. The wild-type strain Dijon was used as a reference. For statistics and conditions, see Figure 1. *** *p* < 0.001; ** *p* < 0.01; * *p* < 0.05.

Next, we targeted subsets of peripheral chemosensory neurons potentially involved in pheromonal perception. Given that “*neur-*GAL4/+, dsUGT36E1/+” males showed reduced CIm, we targeted the dsUGT36E1 transgene in Gr66a gustatory sensory neurons which are involved in the detection of a male aversive pheromone [62,63]. The CIf/CIm performance of transgenic males was not different compared to controls (Figure S3), indicating that UGT36E1 expression in Gr66a-expressing neurons is not required for sex discrimination. Differently, when dsUGT36E1 was targeted in the majority of peripheral olfactory sensory neurons (OSNs) using the *Orco*-GAL4 driver, manipulated males (“*Orco*-GAL4/+, dsUGT36E1/+”) showed a higher discrimination ability as compared to controls (Figure 3A). This effect was due both to (i) the increased CIf of manipulated males compared to *Orco*-Gal4/+ control (*p* = 0.0003) and to (ii) their decreased CIm compared to dsUGT36E1/*+* males (*p* = 0.0034). Note that *Orco*-Gal4/+ control transgenic males, which showed a wild-type-like discrimination, had a significantly decreased sexual activity to target females and/or to target males compared to several other control transgenic males (Figure S4). While the potential alteration induced by GAL4 in some chemosensory tissues has already been reported [62], this finding supports the idea that male sexual activity and sex discrimination can be affected separately. The role of UGT36E1 in sex pheromones detection provides also the first functional evidence of the involvement of an ODE in chemosensory neurons. We cannot exclude the possibility that non-neuronal accessory cells which also express ODEs [64,65] can additionally modulate the male sex pheromone(s) perception. Moreover, our data only provides an indirect evidence of the UGT36E1 expression in head olfactory appendages since we obtained no signal using an antibody specifically designed against this protein.

Given the reciprocal effects induced by the P-UGT36E1 mutation and by the dsUGT36E1 RNAi transgene, both on UGT36E1 mRNA level and sexual discrimination, we combined the two genetic tools in the same fly. Strikingly, “P-UGT36E1/+, *Orco*-GAL4/+, dsUGT36E1/+” males showed a wild- type-like discrimination ability, whereas control “P-UGT36E1/+, dsUGT36E1/+” males showed no such preference (Figure 3A). This indicates that the dsUGT36E1 RNAi (increasing sex discrimination) compensated for the behavioral defect caused by the P-UGT36E1 mutation (decreasing sex discrimination). Moreover, UGT36E1 mRNA levels, measured in the fly appendages, did not differ between “P-UGT36E1/+, *Orco*-GAL4/+, dsUGT36E1/+” males, on one hand and wild-type and transgenic control flies on the other (Figure 3B). These experiments strongly suggest that the gene mutation and the RNAi transgene have additive effects both at the molecular and behavioral levels. 

If we assume that both mRNA and UGT enzyme levels are correlated, our data suggest that the ability of male flies to discriminate sex pheromones depends on the UGT expression level in antennal OSNs. Compared to wild-type males, a reduction of UGT expression level in OSNs tends to increase male ability to discriminate sex pheromones, while a higher level induces the opposite effect. This suggests that the expression level of the UGT gene product in wild-type flies is somewhat intermediate between the levels in the P-UGT36E1 mutant and in “*Orco*-GAL4/+, dsUGT36E1/+” males. Such intermediate level may reflect a trade-off between a relatively high non-specific expression for optimal detoxification and/or signal termination and a relatively low expression allowing wild-type male to acutely detect and discriminate pheromonal stimuli.

**Figure 4:**
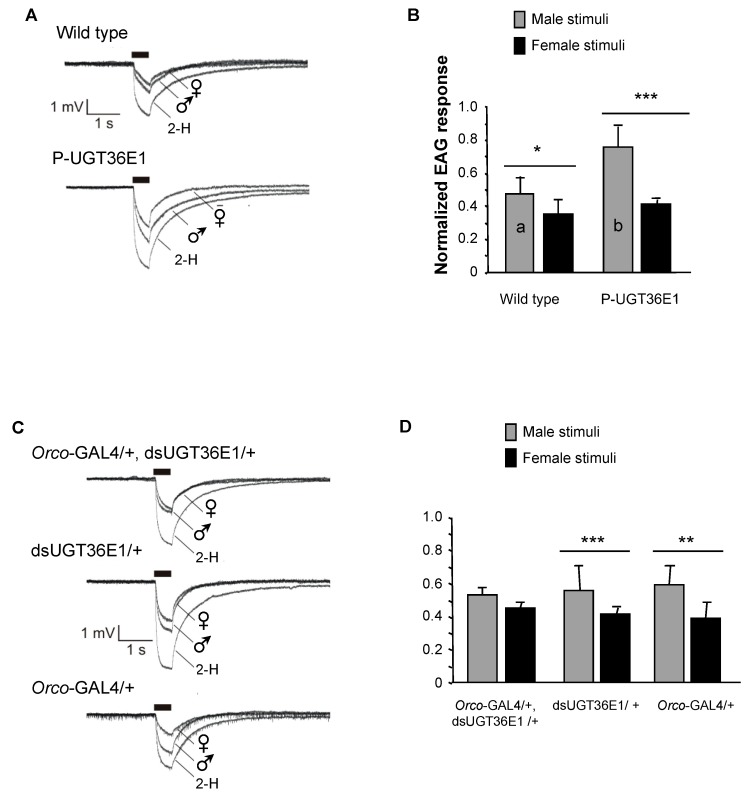
Electrophysiological recording of male olfactory response to different stimuli. (**A**) The graphs represent averaged electroantennogram (EAG) responses of wild-type and P-UGT36E1 mutant male flies to three stimuli: living females, living males and 2-Heptanone (2-H). The thick bars indicate the stimulus duration. (**B**) Bars represent the EAG responses to males and females normalized with the respective responses to 2-H. Statistical differences were noted (i) for the significance of the difference to both sex stimuli (above each pair of bars) and (ii) for responses to male stimuli (letters inside lightly filled bars), between genotypes. (**C**) Averaged EAG responses for “*Orco*-GAL4/+, dsUGT36E1/+”, “dsUGT36E1/+” and “*Orco*-GAL4/+” males presented with the same three stimuli. (**D**) Normalized EAG responses to males and females. “*Orco*-GAL4/+, dsUGT36E1/+” males showed no difference in their responses to either sex. For each test, *n* > 11. For statistics and conditions, see Figure 1. *** *p* < 0.001; ** *p* < 0.01; * *p* < 0.05.

To further investigate the function of UGT36E1 in the peripheral olfaction of sex pheromones, we recorded the global electrophysiological responses of individual male fly antennae (electroantennogram = EAG) either stimulated by male or female volatile pheromonal compounds. We used EAG instead of single sensilla recordings (SSRs) given that we had no idea of the sensillum or sensilla which could respond to the pheromonal mixture. Responses to sex-specific stimuli were normalized with 2-H, a general odorant eliciting robust antennal responses. The responses to these three olfactory stimuli were compared between wild-type, P-UGT36E1 mutant and “*Orco*-GAL4/+, dsUGT36E1/+” males (and in their transgenic parental controls). Wild-type male antenna showed slightly larger relative responses to male than to female volatile compounds (*p* = 0.014; Figure 4A,B). In P-UGT36E1 mutant males, the difference of relative responses to male and female volatile compounds strongly increased (*p* = 0.0006). This increased difference was due to a significantly increased response to male volatile compounds in the mutant compared to wild-type males (*n* = 11–15; *p* = 0.02). However, both mutant and wild-type males showed similar relative responses to female volatile compounds. On the other hand, “*Orco*-GAL4/+, dsUGT36E1/+” experimental males showed similar relative responses to female and male volatile compounds whereas parental transgenic controls (dsUGT36E1/+ and *Orco*-GAL4/+) showed a wild-type-like pattern (e.g., a slightly larger relative response to male than to female volatile compounds; Figure 4C,D). These results indicate that the P-UGT36E1 mutation enhanced the relative amplitude of the electrophysiological response to male volatile compounds whereas the RNAi directed against UGT36E1 in OSNs reduced this response. Therefore, if we cannot formally rule out the possibility that the UGT also affected the EAG response to 2-H, our data clearly show that the manipulation of the UGT gene affected the EAG response to male pheromone(s) relative to female pheromone(s). 

Involvement of ODEs in pheromonal signal modulation was previously hypothesized [20,35,36,66,67,68]. Here we propose that the abundance of the UGT36E1 enzyme in *Drosophila* OSNs can affect the clearance of pheromones in the perireceptor space. Based on this hypothesis, the over-expression of the gene (in P-UGT36E1 mutants) would increase the clearance of the stimulus, promoting faster successive stimulations, and thus enhance the effect of the male inhibitory pheromone (as measured by the EAG), this leading to reduced sex discrimination. Reciprocally, the decreased UGT expression in RNAi targeted males could affect the pheromonal clearance, resulting in an overstimulation which negatively impacts the signal level. This would reduce the aversive effect induced by the male pheromone(s) and increase male ability to discriminate sex pheromones. Based on these observations, we hypothesize that decreased pheromonal clearance in the perireceptor space promoted the saturation of dedicated receptors and reduced both OSN sensitivity and relative EAG amplitude. In support of our interpretation, two other studies based on the detection of different chemical or use of different genetic tools, reported that the alteration of activity in phase I enzymes (CYP or CES) also induced a prolonged olfactory neuronal response leading to an altered perception of the pheromones highlighting their role in signal termination [7,36].

How can we explain the apparent conundrum between the increased EAG response to male pheromone and the decreased discrimination shown by mutant males (and the reciprocal effects in RNAi targeted males)? In our behavioral assay, wild-type male OSNs were simultaneously stimulated by a mixture of inhibitory and attractive olfactory pheromones emitted by male and female flies, respectively. We hypothesize that the increased nervous antennal response to male inhibitory pheromone(s) in the perireceptor space may disturb the response to female pheromone(s), and this unbalanced effect would affect their integrated comparison in brain structures which are normally involved in the sex pheromone discrimination [69,70]. In any case, our data reveal that this mechanism likely depends on the level of the UGT gene product: males combining both the mutation and the RNAi targeted in neural tissues showed a wild-type-like behavioral discrimination. This is reminiscent of a recent transcriptomic study performed in the *Bombyx mori* silkworm antenna which revealed that the olfactory impairment observed in the domestic strain is correlated with a decreased expression of ODEs (including some UGTs) as compared to the wild *B. mori* strain [15]. 

In summary, our data reveal that the reciprocal variation of a UGT expression can change male sex discrimination in opposite directions. This effect is likely based on the ability of manipulated flies to discriminate between volatile sex pheromones. Given the high diversity, the ubiquitous distribution, the regulation and the varied properties of ODEs in animals, our findings provide a significant step to unravel the complexity of mechanisms underlying olfactory sensitivity at the peripheral nervous system. 

## Supplementary Materials

The following are available online at https://www.mdpi.com/2073-4425/11/3/237/s1, Figure S1: Expression ratio of UGT36E1 in appendages, head and thorax and abdomen, Figure S2: Locomotor activity tests, Figure S3: Effect of dsUGT36E1 targeted in Gr66a gustatory neurons, Figure S4: Male courtship in control strains.
