# Supplementary material for: Modulation of Sex Pheromone Discrimination by a UDP-Glycosyltransferase in Drosophila melanogaster
Source: Genes (Basel). 2020 Feb 25;11(3):237. doi: 10.3390/genes11030237 (PMC7140800; doi:10.3390/genes11030237)
Supplement: Supplementary file 1 [file genes-11-00237-s001.zip › genes-717705-supplementary.pptx]

## Slide 1
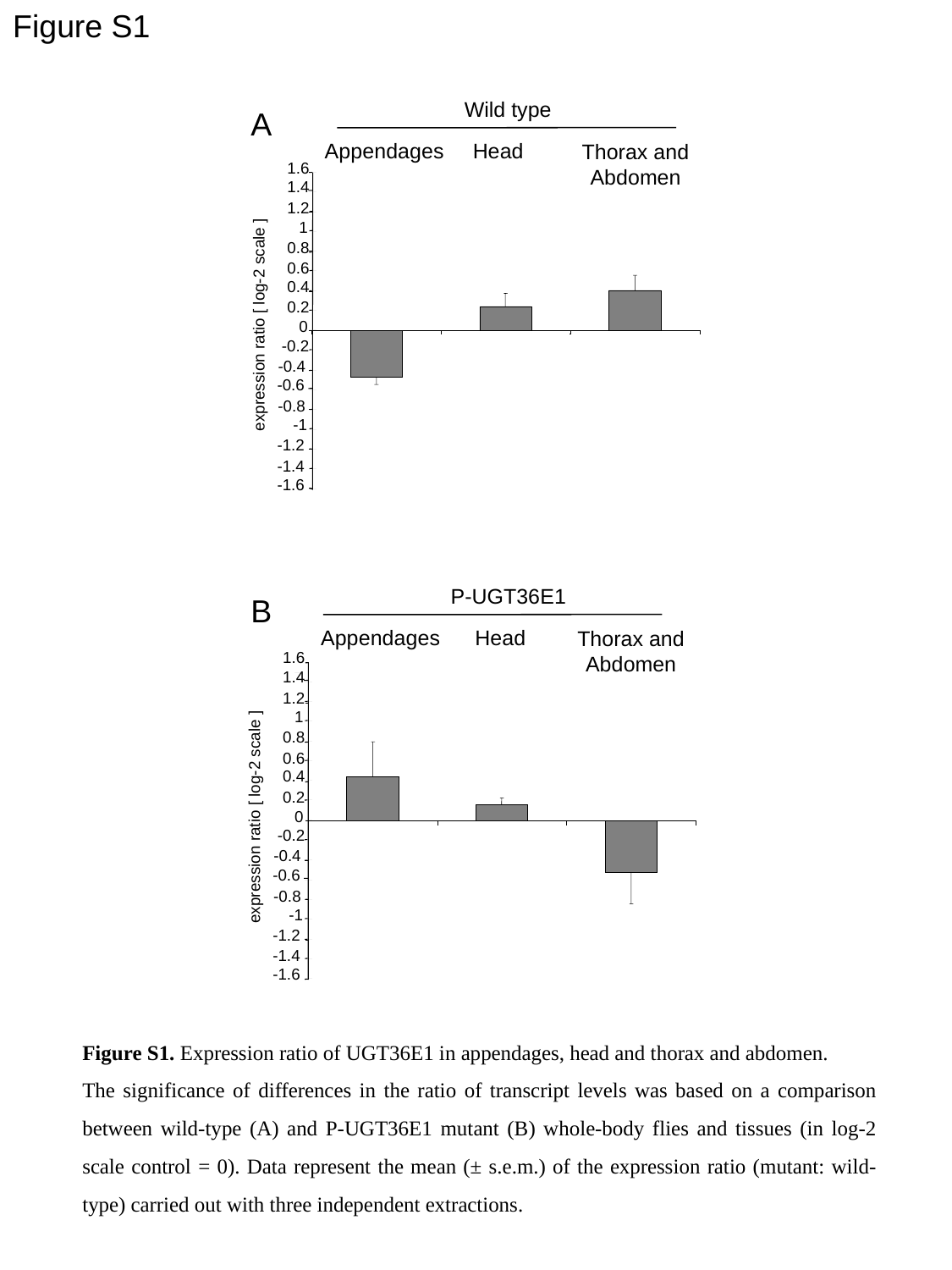

Figure S1
Wild type
A
Appendages
Head
Thorax and Abdomen
1.6
1.4
1.2
1
0.8
0.6
0.4
0.2
expression ratio [ log-2 scale ]
0
-0.2
-0.4
-0.6
-0.8
-1
-1.2
-1.4
-1.6
P-UGT36E1
B
Appendages
Head
Thorax and Abdomen
1.6
1.4
1.2
1
0.8
0.6
0.4
0.2
expression ratio [ log-2 scale ]
0
-0.2
-0.4
-0.6
-0.8
-1
-1.2
-1.4
-1.6
Figure S1. Expression ratio of UGT36E1 in appendages, head and thorax and abdomen.
The significance of differences in the ratio of transcript levels was based on a comparison between wild-type (A) and P-UGT36E1 mutant (B) whole-body flies and tissues (in log-2 scale control = 0). Data represent the mean (± s.e.m.) of the expression ratio (mutant: wild-type) carried out with three independent extractions.

## Slide 2
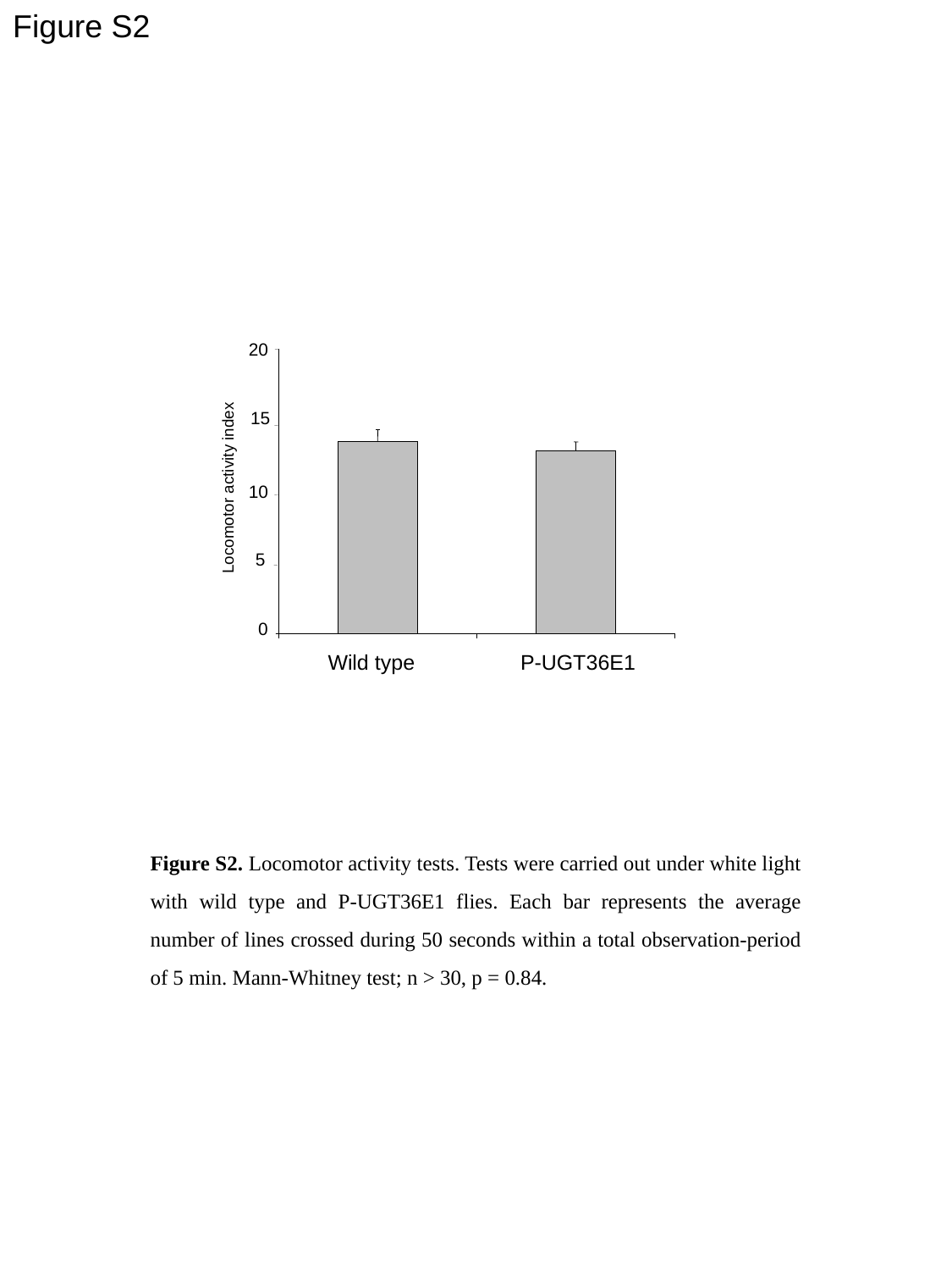

Figure S2
20
15
Locomotor activity index
10
5
0
Wild type
P-UGT36E1
Figure S2. Locomotor activity tests. Tests were carried out under white light with wild type and P-UGT36E1 flies. Each bar represents the average number of lines crossed during 50 seconds within a total observation-period of 5 min. Mann-Whitney test; n > 30, p = 0.84.

## Slide 3
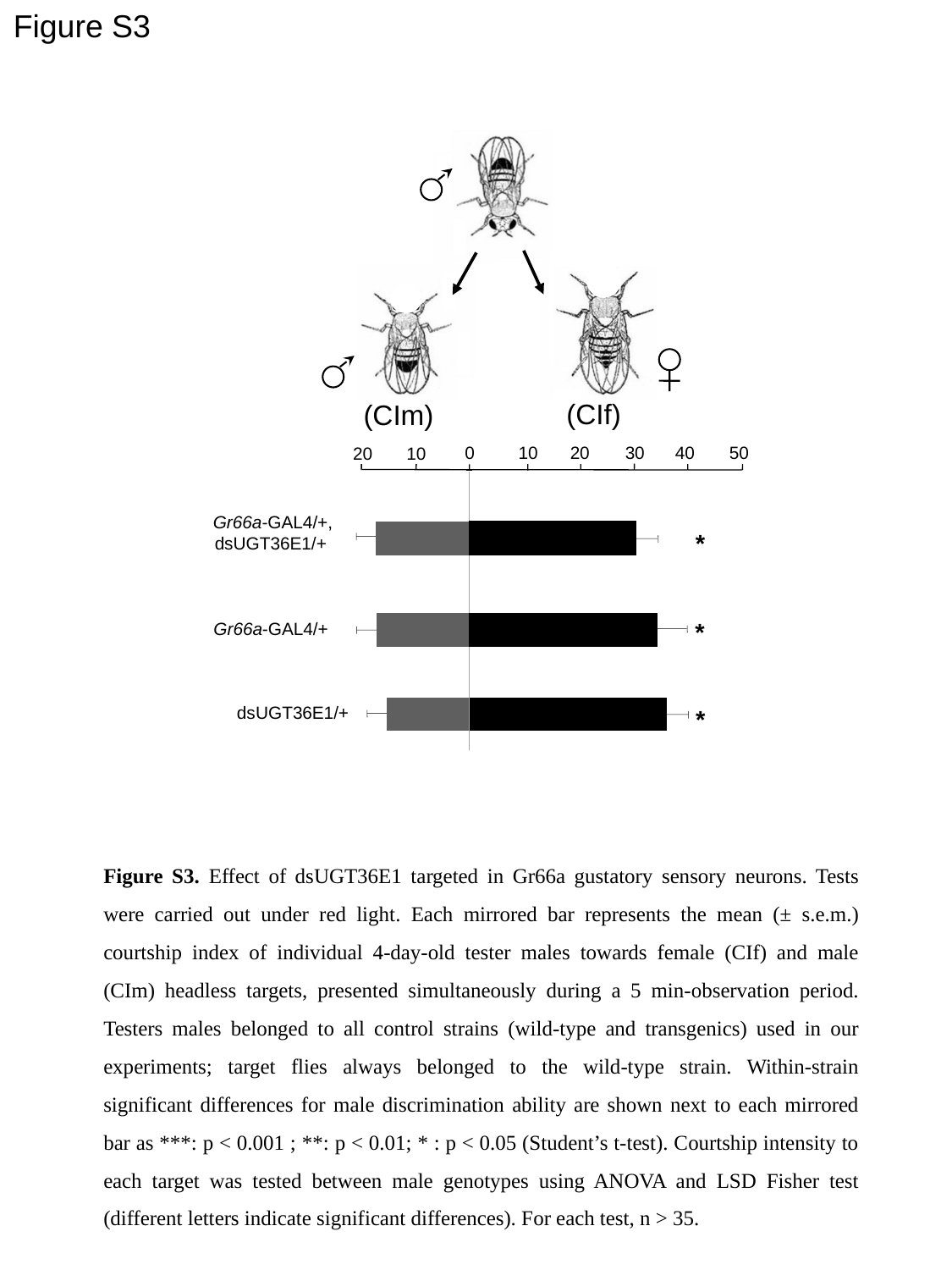

Figure S3
(CIf)
(CIm)
20
40
50
10
0
30
20
10
Gr66a-GAL4/+, dsUGT36E1/+
*
*
Gr66a-GAL4/+
dsUGT36E1/+
*
Figure S3. Effect of dsUGT36E1 targeted in Gr66a gustatory sensory neurons. Tests were carried out under red light. Each mirrored bar represents the mean (± s.e.m.) courtship index of individual 4-day-old tester males towards female (CIf) and male (CIm) headless targets, presented simultaneously during a 5 min-observation period. Testers males belonged to all control strains (wild-type and transgenics) used in our experiments; target flies always belonged to the wild-type strain. Within-strain significant differences for male discrimination ability are shown next to each mirrored bar as ***: p < 0.001 ; **: p < 0.01; * : p < 0.05 (Student’s t-test). Courtship intensity to each target was tested between male genotypes using ANOVA and LSD Fisher test (different letters indicate significant differences). For each test, n > 35.

## Slide 4
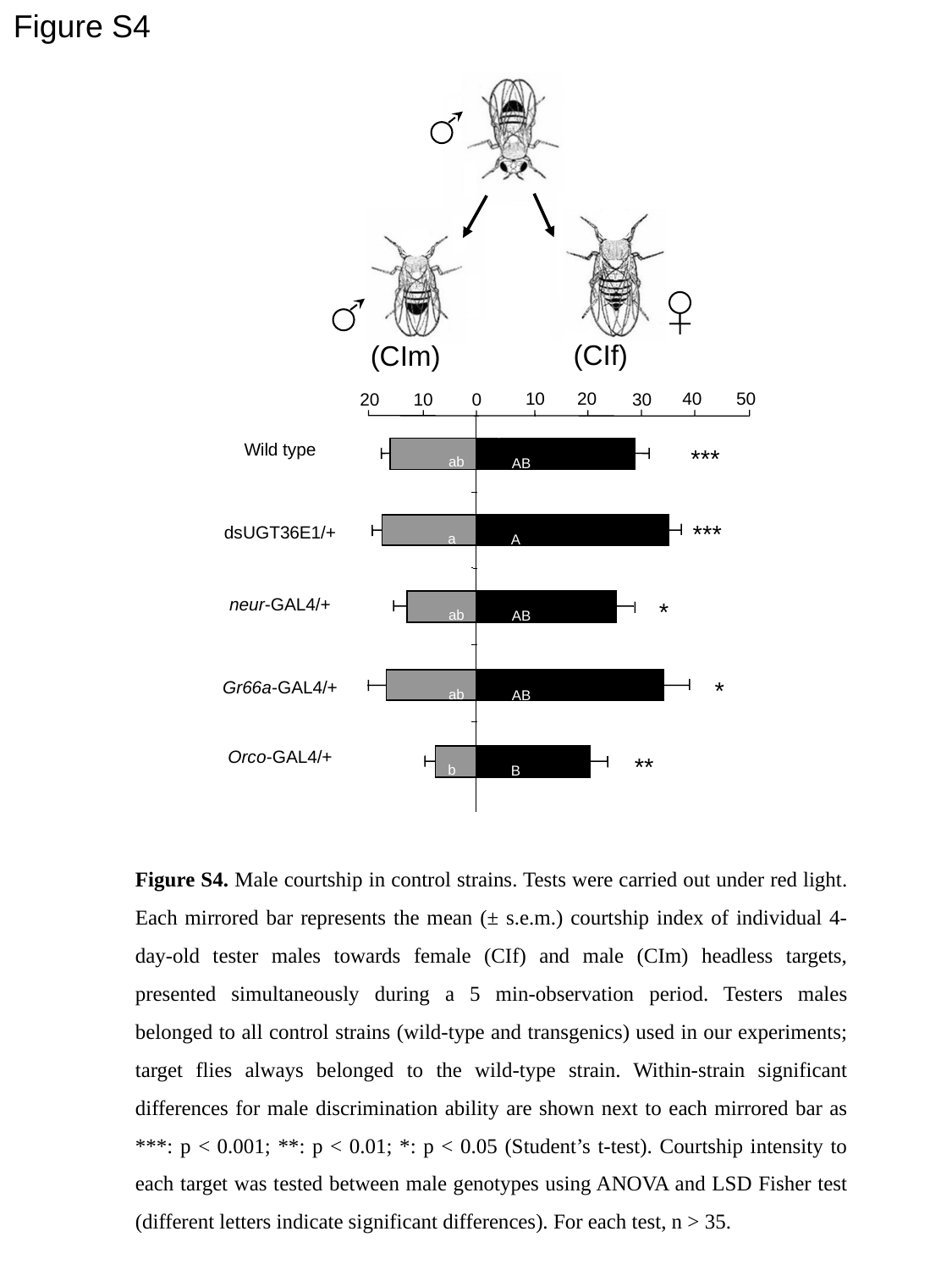

Figure S4
(CIf)
(CIm)
20
40
50
10
0
30
20
10
Wild type
ab
AB
***
a
A
***
dsUGT36E1/+
ab
AB
neur-GAL4/+
*
ab
AB
*
Gr66a-GAL4/+
b
B
Orco-GAL4/+
**
Figure S4. Male courtship in control strains. Tests were carried out under red light. Each mirrored bar represents the mean (± s.e.m.) courtship index of individual 4-day-old tester males towards female (CIf) and male (CIm) headless targets, presented simultaneously during a 5 min-observation period. Testers males belonged to all control strains (wild-type and transgenics) used in our experiments; target flies always belonged to the wild-type strain. Within-strain significant differences for male discrimination ability are shown next to each mirrored bar as ***: p < 0.001; **: p < 0.01; *: p < 0.05 (Student’s t-test). Courtship intensity to each target was tested between male genotypes using ANOVA and LSD Fisher test (different letters indicate significant differences). For each test, n > 35.
